# Fracture-Related Infection of the Lower Limb Caused by Mucor velutinosus: Amputation or Salvation?

**DOI:** 10.7759/cureus.65988

**Published:** 2024-08-02

**Authors:** Alexander Eijkenboom, Matthias Militz, Maurizio Papetti, Veit Krenn, Simon Hackl

**Affiliations:** 1 Department of Septic and Reconstructive Surgery, BG Unfallklinik Murnau, Murnau am Staffelsee, DEU; 2 Department of Pathology, MVZ Institute of Pathology, Trier, DEU; 3 Institute of Biomechanics, BG Unfallklinik Murnau, Murnau am Staffelsee, DEU

**Keywords:** fungal infection, ankle arthrodesis, amphotericin b, fracture-related infection, mucor velutinosus

## Abstract

Fracture-related infections caused by mucormycosis are rare and potentially fatal. Evidence-based experience with its treatment is limited, and surgical management ranges from limb salvage to amputation, with indications not always clear. A 56-year-old woman was admitted after an aircraft accident, sustaining major trauma injuries, including a Gustilo-Anderson type III open ankle joint fracture. Initial damage control surgery with external fixation ensued, followed by secondary, definitive internal fixation with plate and screws. The patient developed a fracture-related infection in the ankle caused by *Mucor velutinosus*. Despite its invasive growth and tenacity, surgical debridement combined with systemic and local antifungal therapy led to remission in this immunocompetent patient. The ankle arthrodesis achieved bone union with a hexapod fixator 10 months post-trauma. In the treatment of opportunistic invasive mucormycosis, a multidisciplinary approach is necessary, especially in patients suffering major trauma injuries. Through apt diagnosis and thorough treatment by experienced surgeons, infectiologists, and pathologists, successful limb salvage may be attained in patients with an otherwise intact immune system, and amputation can be prevented.

## Introduction

Mucormycosis is a rare angioinvasive fungal infection caused by filamentous fungi such as *Rhizopus species*, *Mucor species*, and *Lichtheimia species*, with a high morbidity and a mortality rate ranging from 40% to 80% [1]. Mucor spores are ubiquitous in nature [2]. Usually, manifestations were observed as rhino-orbital-cerebral mucormycosis, followed by cutaneous and pulmonary mucormycosis [2,3]. Mucormycosis typically affects immunocompromised patients with underlying conditions such as diabetes mellitus, hematologic malignancies, organ transplants, and neutropenia [1,2]. It has also become a new hazard in the treatment of patients with COVID-19 who were receiving high-dose corticosteroids [4]. Osteoarticular infections caused by fungi are extremely rare and are mostly associated with Candida or Aspergillus [5].

The following case report was previously posted to the Research Square preprint server on April 29, 2024.

## Case presentation

**Figure 1:**
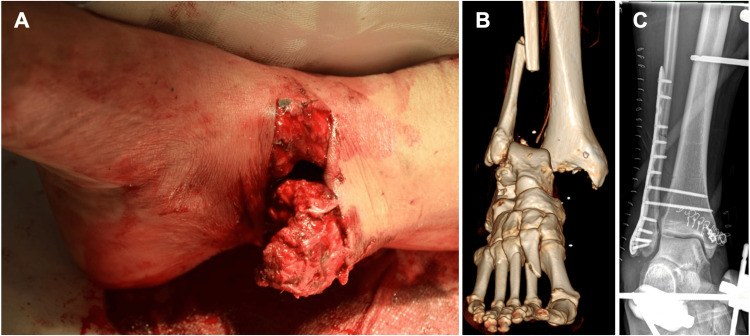
Type III open ankle joint fracture (A) Photograph of the open ankle joint fracture with protruding medial malleolus through the skin at admission. (B) 3D rendered image from a CT scan performed at admission, visualizing a distal fracture of the fibula and dislocation of the tibiotalar joint. (C) Osteosynthesis of the fibula by plate and reconstruction of the deltoid ligament by suture anchor with additional external fixation.

A 56-year-old female with no medical history apart from cervical spinal canal stenosis and hypothyroidism was admitted following a light aircraft crash. A contrast-enhanced whole-body computer tomography (CT) scan was performed, revealing major trauma injuries, including fractures of the cervical, thoracic, and lumbar spine, bilateral rib fractures, and a sternal fracture. Additionally, a Gustilo-Anderson type III open ankle joint fracture of the right lower extremity was detected (Figures 1A, 1B).

Intravenous antibiotics with ampicillin/sulbactam (2 g/1 g three times daily) were initiated preoperatively and continued for 72 hours due to the type III open fracture. Following admission, immediate reduction of the upper ankle joint and stabilization by external fixation were performed in accordance with damage control principles. The soft tissue injury was surgically debrided, tissue samples for culture were taken, and gentamicin-impregnated collagen fleece was applied. Negative pressure wound therapy (NPWT) was used for temporary wound closure. Four days after trauma, the patient underwent anterior cervical disc fusion and internal fixation of the thoracolumbar junction spine fracture.

Following surgery, the patient developed hospital-acquired pneumonia, which required escalation of antibiotic treatment to piperacillin/tazobactam (4.5 g four times daily). After seven days, pulmonary function recovered sufficiently, and antibiotic therapy was discontinued. The lower limb fracture was definitively treated through open reduction and internal fixation on day 10. A locking plate was used for the distal fibula, along with tibiofibular syndesmotic screw fixation and a suture anchor for deltoid ligament reconstruction (Figure 1C). Additionally, the medial wound was closed with staples during the same surgery, while the external fixator remained to aid in soft tissue healing. At this point, the tissue cultures have not shown any bacterial or fungal growth. On day 16, the external fixator was removed.

**Figure 2:**
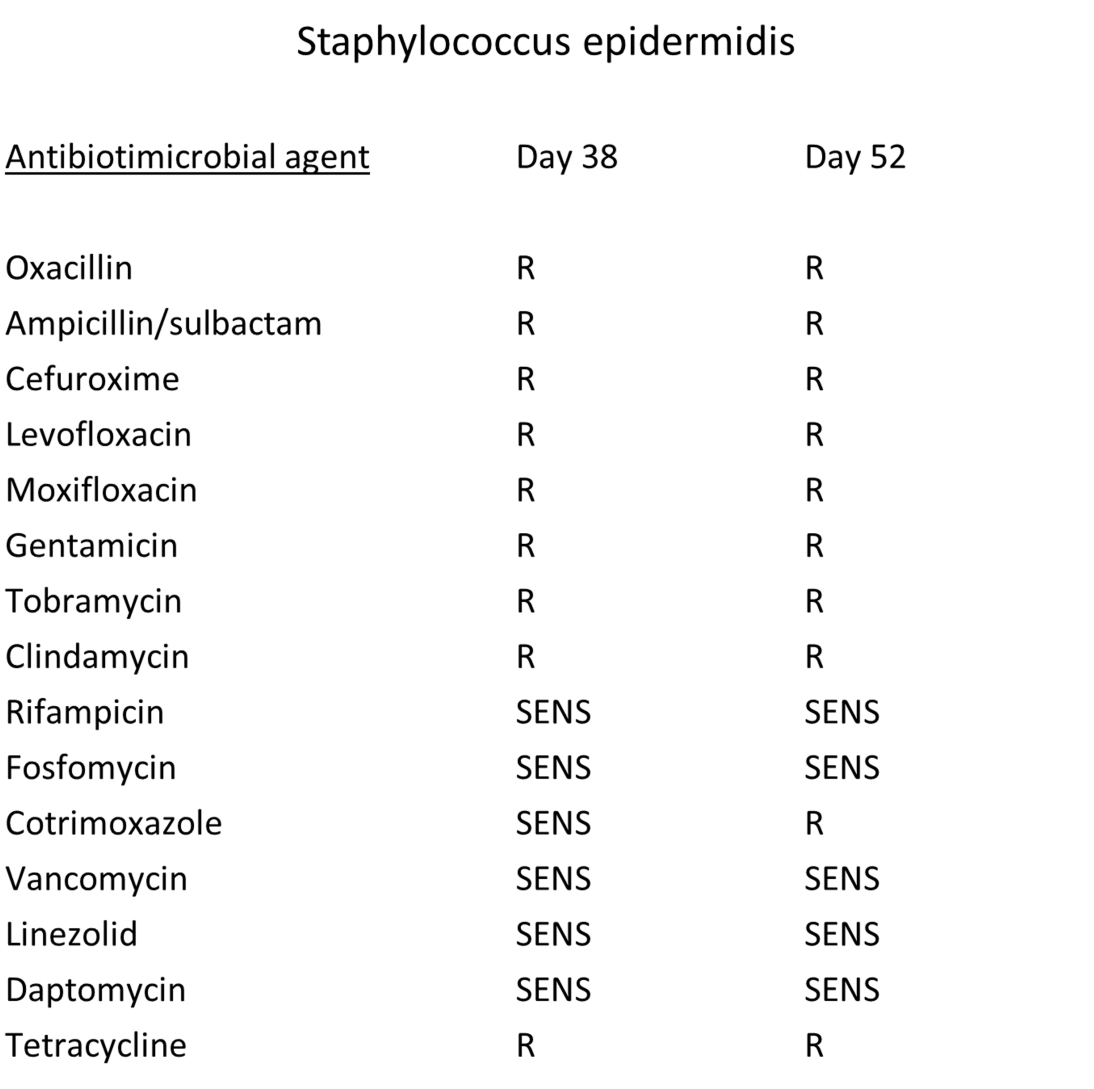
Antibiotic sensitivity testing of Staphylococcus epidermidis Resistance (R) and sensitivity (SENS) of detected *Staphylococcus epidermidis* from tissue samples taken on days 38 and 52.

On day 38, a surgical wound revision was performed due to delayed healing and continuous serosanguinous drainage of the medial ankle wound. The revision included debridement of the medial malleolus, taking tissue samples, and lavage of the upper ankle joint, as well as an intraoperative demonstration of the surgical findings to our plastic surgeons. A fracture-related infection (FRI) of the distal tibia was diagnosed, as the confirmatory criteria for FRI were fulfilled with the presence of a fistula connecting to the medial malleolus and implants. Any growing microorganisms were analyzed using matrix-assisted laser desorption ionization-time of flight mass spectrometry (MALDI-TOF MS). Upon detection of *Staphylococcus epidermidis* (*S. epidermidis*) with MALDI-TOF MS in all tissue samples, antibiotic sensitivity testing was conducted. Cotrimoxazole (960 mg twice daily) was administered orally. The patient underwent repeated surgical debridement, including the removal of the suture anchor, resulting in a growing soft tissue defect at the medial malleolus. The antibiotic therapy was changed to linezolid (600 mg twice daily) due to a change in the antibiotic sensitivity of the cultured *S. epidermidis* (Figure 2).

**Figure 3:**
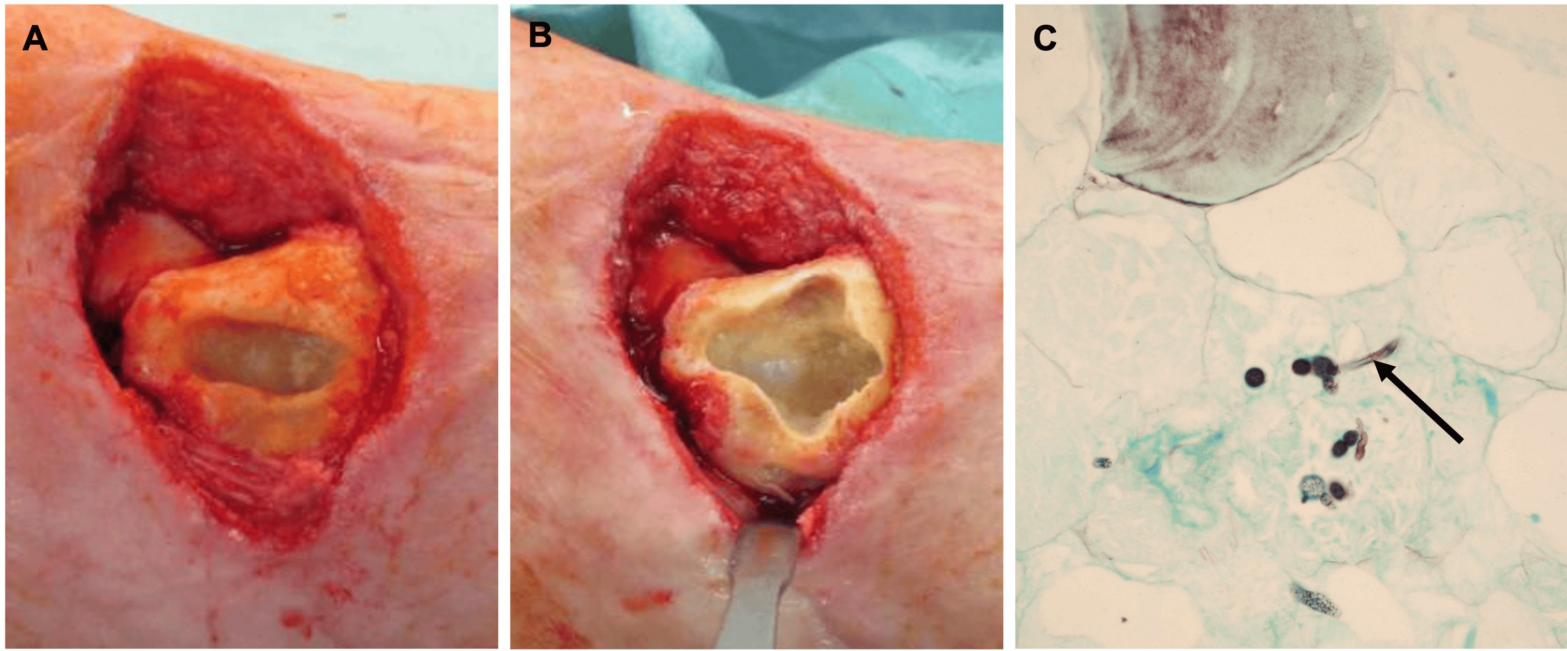
Mucormycosis of bone tissue (A) Avital medial malleolus and soft tissue defect after removal of the suture anchor. (B) Medial malleolus after surgical debridement. The cancellous bone shows no bleeding. (C) Microscopic image (420×) of the necrotic bone. Grocott's methenamine silver stain shows hyphae of *M. velutinosus* (arrow).

On day 65, after surgical debridement and tissue sampling, the presence of fungi was first detected in cultures. The fungus was classified as *Mucor velutinosus* (*M. velutinosus*) using MALDI-TOF MS. Hyphae were identified in histopathology. Furthermore, the tibia exhibited necroses with a lack of cancellous bone bleeding (Figure 3). Antifungal therapy for osseous mucormycosis was initiated with posaconazole (600 mg the first day, then 300 mg daily). Systemic inflammatory markers were almost normal (C-reactive protein (CRP) 1.7 mg/dL, cut-off <1.0 mg/dL; white blood cell (WBC) 7.5/nL), and the patient presented stable.

During subsequent debridement surgeries, positive cultures for *M. velutinosus* and *S. epidermidis* were found in biopsies of the upper ankle and distal fibula. All foreign implants in the distal fibula were removed on day 70. The upper ankle joint showed a chronic FRI and septic cartilage destruction. A CT angiography revealed sufficient arterial perfusion of the lower extremities without relevant stenoses.

Two therapeutic options were discussed with the patient. The first option was below-knee amputation for rapid recovery and weight-bearing activities, assuming proper wound healing. The second option was limb salvage, including ongoing antibiotic and antifungal therapy, debridement, bone reconstruction, and soft tissue reconstruction under continuous clinical monitoring. The concept of limb salvage was considered feasible for an immunocompetent, stable patient. Limb salvage was chosen according to the patient's wishes.

**Figure 4:**
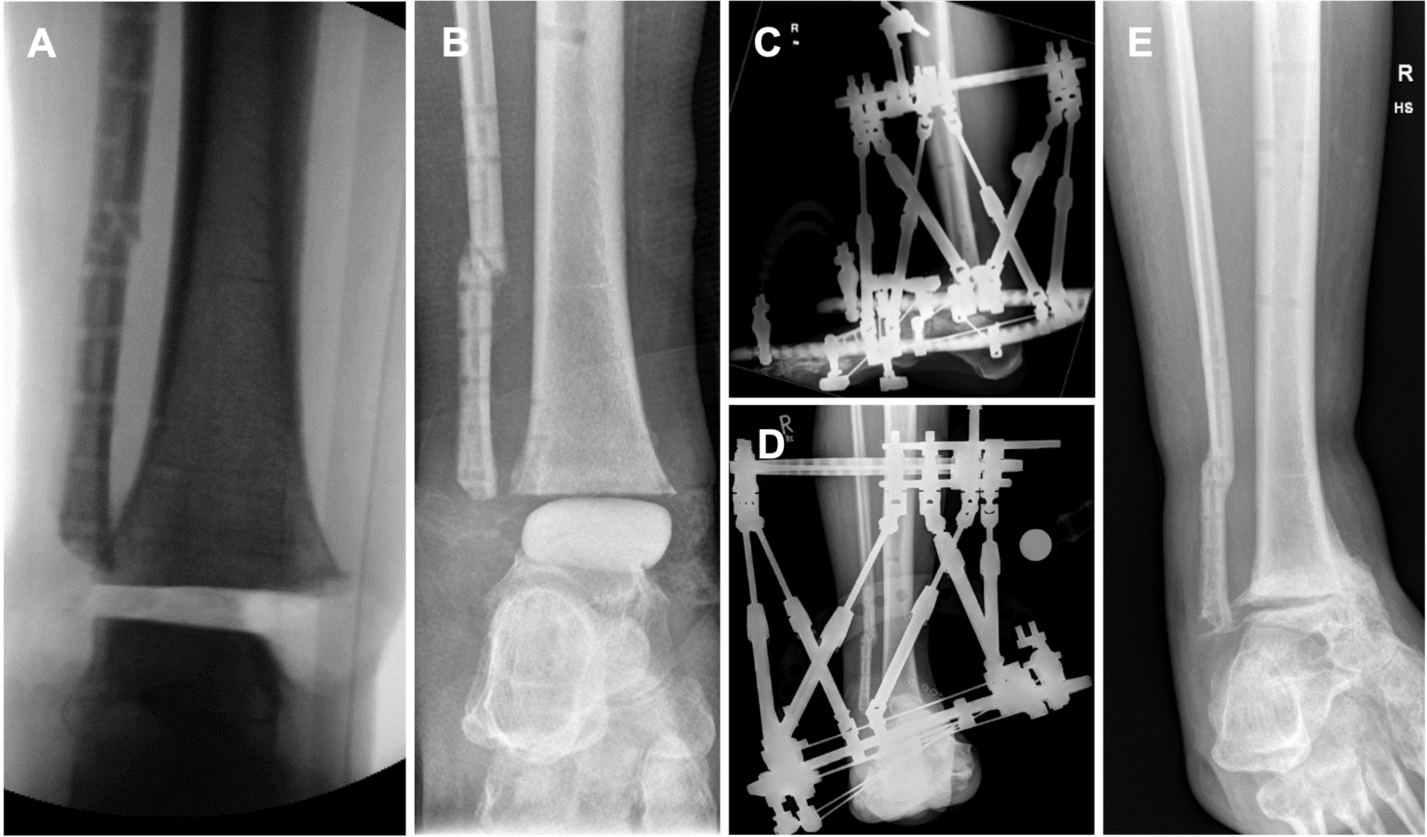
Radiographs of the lower leg (A) Resection of the upper ankle joint, including the medial and lateral malleolus, and removal of all foreign implants. (B) Amphotericin B and vancomycin-infused PMMA spacer during the debridement phase. (C, D) Hexapod fixator to correct varus deformity. (E) Partial bone union after removal of the hexapod fixator.

On day 80, the upper ankle joint surfaces, specifically the medial and lateral malleolus, were resected (Figure 4A). Temporary bone defect management for the former upper ankle joint was achieved using malleable polymethylmethacrylate (PMMA) cement spacers impregnated with amphotericin B (AmB, 200 mg in 20 g PMMA) and vancomycin (1 g in 20 g PMMA) (Figure 4B). Additionally, the antifungal treatment was escalated to intravenous liposomal AmB (250 mg daily, 3-5 mg/kg body weight daily). Weekly debridement was repeated for five weeks until *M. velutinosus* and *S. epidermidis* were no longer detectable in tissue samples, and the resection margin was microscopically confirmed to be free of mucor hyphae. As a result of these debridement surgeries, a 5 cm bone defect, as well as a medial soft tissue defect, remained. Linezolid was discontinued.

A Hoffmann Limb Reconstruction Frame (LRF) hexapod fixator (Stryker®, Kalamazoo, Michigan) was applied on day 114 for arthrodesis of the upper ankle joint after final bone debridement. The considerable soft tissue defect required limb shortening and fixation of the foot in a varus position of 30° so that successful secondary wound closure could be performed together with plastic surgery (Figures 4C, 4D). This approach circumvented the need for extensive reconstruction, as trauma and plastic surgeons were both reluctant to perform a free flap transplantation in a mucormycotic FRI. The wound consolidated after three weeks, allowing earlier mobilization than with a transplanted free flap. Intravenous liposomal AmB was discontinued on day 127, four weeks after initiation. The hexapod fixator was used to continuously correct the combined bony and soft tissue deformity over a period of 50 days, finishing on day 180.

**Figure 5:**
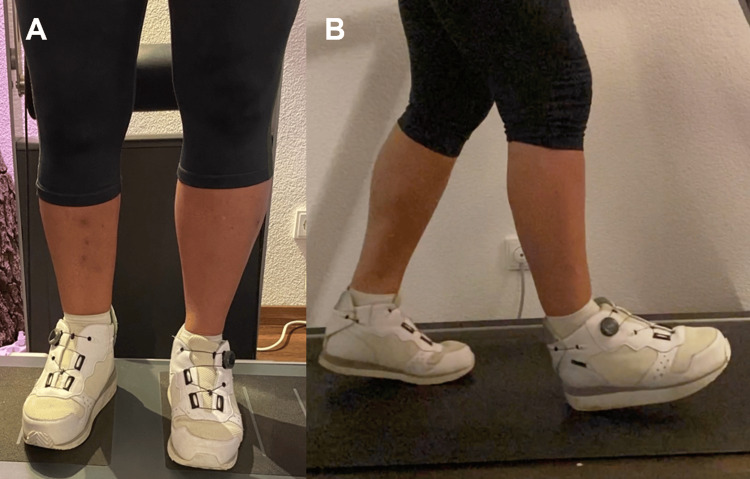
Clinical presentation one year post-trauma (A) Anterior view showing reduced muscle mass of the right lower limb after ankle arthrodesis. (B) Lateral clinical view showing the shoe lift.

A neutral position of the tibiotalar arthrodesis was achieved with sufficient and stable soft tissue coverage. Routine clinical examinations did not reveal signs of infection, and radiological imaging indicated progressing bone union. The LRF fixator was removed after six months. The healing arthrodesis was further immobilized using a walking boot orthosis. Physical therapy was intensified, and the patient was able to walk without pain. Radiological imaging showed partial but sufficient union of the ankle arthrodesis 10 months after the reconstruction surgery (Figure 4E). The patient currently exhibits a healthy gait and posture by compensating for the leg length discrepancy with a minor shoe lift (Figure 5). No further surgical treatment is planned due to the patient's lack of pain and good mobility. She has no regrets regarding the mutual decision for limb salvation.

## Discussion

Interestingly, our patient did not suffer from pre-existing medical conditions, which makes an FRI caused by *M. velutinosus* even more remarkable. However, a meta-analysis has shown that major trauma may increase the risk of mucormycosis [3]. In the case of our otherwise healthy patient, mucormycotic FRI likely developed as a consequence of post-injury immunosuppression following major trauma. The origin of *M. velutinosus* in our patient remains unclear. Taj-Aldeen et al. suggested that 56% of these infections are caused by direct inoculation during trauma, 24% by hematogenous dissemination, and 21% by contiguous spread [6]. In their study, the median diagnostic delay for mucormycosis was 60 days. This is reflected in our case, in which *M. velutinosus* was first detected on day 65. Direct inoculation in a type III open ankle joint fracture seems most likely.

The osseous mucormycosis of our patient was classified as an FRI since the pathogenic bacteria and fungi were present at the fracture site and implants [7]. Hereby, FRIs, or any osteoarticular infections caused by fungi, are extremely rare and are mostly associated with Candida or Aspergillus [5]. Osseous mucormycosis remains an exception in an already rare disease. Current literature only exists regarding individual case reports of immunocompromised patients [6,8].

Case reports of mucormycotic FRI are rare, and different treatment regimens are described [6,8]. Treatment suggestions included surgical debridement, systemic antifungal therapy, and often amputation of affected lower limbs [9,10]. Case reports with limb salvage in well-vascularized body regions such as the hand have also been reported [11]. Amputation was performed in more instances than not and seemed a possibility for our patient as well, considering the invasiveness of mucormycosis with bone and soft tissue destruction leading to high mortality [6,9,10,12].

After diagnosing FRI in our patient, we initiated anti-infective therapy, including surgical debridement, removal of foreign implants, and adjuvant systemic antimycotics with oral posaconazole. Due to our patient's immunocompetence and lack of medical history, we extensively discussed possible treatment options with the patient. Eventually, we pursued the concept of limb salvage under close clinical observation. After six weeks of unsuccessful eradication of *M. velutinosus* with posaconazole, the guidelines of the European Society of Clinical Microbiology and Infectious Diseases recommend escalation to intravenous liposomal AmB for the treatment of invasive fungal infections [13]. An earlier escalation may have led to the earlier eradication of the infection. However, it is important to note that AmB can lead to serious adverse effects, such as nephrotoxicity [14,15]. Due to the absence of acute signs of infection, an escalation was initially waived.

Our therapy also involved the local application of AmB using PMMA spacers to achieve higher antifungal concentrations on-site. PMMA spacers are malleable, can be adapted to the osseous defect, and also allow easy removal when compared to other temporary bone void fillers. This method has been proven to be highly effective in treating bacterial infections in cases where poorly vascularized bone is not accessible to systemic intravenous anti-infective therapy [16,17].

Hyperbaric oxygenation therapy (HBOT) can be considered an additional therapeutic tool for treating mucormycosis. Data on the effectiveness of HBOT in treating fungal infections are limited and inconsistent. HBOT has been suggested to promote wound healing and inhibit fungal growth, and it has been proposed as an adjunct therapy to antifungal and surgical treatments for rhinocerebral mucormycosis [18,19].

In this case, radical debridement of the upper ankle joint combined with antifungal medication resulted in the eradication of the fungal infection. This procedure resulted in a bone defect of approximately 5 cm. Our interdisciplinary team discussed various reconstruction options, including internal fixation, allograft or autograft bone transplants, external fixation, and soft tissue reconstruction with a free flap. Ultimately, the multidisciplinary team decided against free flap surgery, given the limited experience with invasive mucormycotic FRI and the risk of an ongoing infection potentially resulting in complications at the free flap donor site. Due to the risk of infection relapse, a hexapod fixator was chosen as a reliable external fixation method for complex lower limb injuries [20]. Limb salvage was ultimately achieved, with a follow-up of 12 months post-trauma.

In the era of standard operating procedures and algorithms, the authors suggest the following approach for immunocompetent patients with mucormycotic FRI: treatment by an experienced team of surgeons and infectiologists is recommended. Tissue samples should also be examined for fungal pathogens using cultures and histopathology. After pathogen identification and diagnosis of mucormycotic FRI, antifungal treatment with oral posaconazole or, if possible, intravenous liposomal AmB is recommended. Early and complete removal of foreign implants, as well as necrotic tissue, is inevitable during the debridement of soft tissue and bone. Local antifungal treatment options, such as AmB-infused PMMA spacers, can be utilized. Adjunct HBOT may be considered. Reconstruction of soft tissue and bone can be performed once tissue samples test negative and the ongoing FRI is eliminated. Temporary post-injury immunosuppression and the recovery of the patient after this play a key role in regard to the available treatment options.

In cases of rapid progression of mucormycosis or lack of reconstructive options, the treatment course of limb salvage may be aborted, with amputation available as a fallback.

## Conclusions

Opportunistic mucormycotic FRI may be a result of post-injury immunosuppression after major trauma. Multidisciplinary cooperation is crucial for salvaging the lower extremity in cases of mucormycotic FRI. This begins with early histopathological and microbiological examinations, which allow for timely surgical and antifungal treatment. Despite the invasive growth of mucormycetes, it is important to avoid hasty decisions regarding amputation, especially in patients with no comorbidities and an otherwise intact immune system. Limb salvage is a viable option in a setting where ongoing clinical reevaluation is possible and immediate surgical intervention is available in case of infection aggravation.
